# The effect of nicorandil in patients with cardiac syndrome X: A meta-analysis of randomized controlled trials

**DOI:** 10.1097/MD.0000000000022167

**Published:** 2020-09-11

**Authors:** Qiulei Jia, Shuqing Shi, Guozhen Yuan, Jingjing Shi, Shuai Shi, Yi Wei, Yuanhui Hu

**Affiliations:** a Department of cardiovascular, Guang’anmen Hospital, China Academy of Chinese Medical Sciences, Beijing; b Graduate School, Beijing University of Chinese Medicine, China.

**Keywords:** cardiac syndrome X, meta-analysis, nicorandil, randomized controlled trials, systematic review

## Abstract

**Background:**

The prevalence of cardiac syndrome X (CSX) is considerable. Some patients show recurrent angina attacks and have a poor prognosis. However, the knowledge of CSX pathophysiological mechanism is still limited, and the treatment fails to achieve a satisfactory suppression of symptoms. Nicorandil has a beneficial effect on improving coronary microvascular dysfunction (CMD). This study aims to evaluate the clinical effects and safety of nicorandil on CSX patients.

**Methods:**

The Cochrane Library, Pubmed, EMBASE, ClinicalTrials.gov and 4 Chinese databases were searched to identify relevant studies. The Cochrane “Risk of bias” tool was used to assess the methodological quality of eligible studies. Meta-analysis was performed by RevMan 5.3 software. The Eggers test and meta-regression were performed by software Stata 14.0. Quality of evidence was assessed using the Grading of Recommendations Assessment, Development and Evaluation (GRADE) approach.

**Results:**

Twenty four randomized controlled trials (RCTs) involving 2323 patients were included. Most of the included studies were classified as having an unclear risk of bias because of poor reported methodology. The main outcomes are angina symptoms improvement, resting electrocardiogram (ECG) improvement, treadmill test result, and endothelial function. Meta-analysis showed that nicorandil had some benefit on improving angina symptoms (RR 1.24, 95% CI 1.19 to 1.29, *I*^2^ = 20%, *P* < .00001), resting ECG (RR = 1.24, 95% IC: 1.15 to 1.33, *I*^2^ = 0%, *P* < .00001), and prolonged the time to 1 mm ST-segment depression in treadmill test result (WMD = 38.41, 95% IC: 18.46 to 58.36, *I*^2^ = 0%, *P* = .0002). Besides nicorandil could reduce the level of endothelin-1 (ET-1) (SMD = −2.22, 95% IC: −2.61 to −1.83, *I*^2^ = 77%, *P* < .00001) and increase the level of nitric oxide (NO) (WMD = 27.45, 95% IC: 125.65 to 29.24, *I*^2^ = 81%, *P* < .00001). No serious adverse drug event was reported. The Eggers test showed that significant statistical publication bias was detected (Eggers test *P *= .000). The quality of evidence ranged from very low to low.

**Conclusions:**

Nicorandil shows the potential of improving angina symptoms, ECG, and endothelial dysfunction in patients with CSX. However, there is insufficient evidence for the clinical benefits of nicorandil due to the very low-quality evidence.

## 1 Introduction

Cardiac syndrome X (CSX) is usually described as patients with effort-induced symptoms similar to those observed in patients with angina triggered by obstructive coronary artery disease (CAD), poor, or slow response to nitroglycerin, objective evidence of myocardial ischemia including the abnormal electrocardiogram (ECG) and/or stress test results, and completely normal or near-normal coronary arteriograms. No cardiac or systemic diseases should be detectable in these patients.^[1]^ The symptom of CSX is regarded as microvascular angina (MVA).^[2]^

Data from the National Cardiovascular Data Registrys CathPCI Registry showed that nearly 60% of 661,063 patients undergoing elective coronary angiography had normal coronary arteries or non-obstructive CAD (stenoses <50%).^[3]^ Coronary microvascular dysfunction (CMD) is typically the mechanism underlying CSX, which refers to impaired vasodilatation and/or increased sensitivity to vasoconstriction in the small resistance coronary arteries.^[1,4]^ Another study certified two-thirds of patients presenting with chest pain in the absence of obstructive CAD showed evidence of microvascular dysfunction.^[5]^

Previous studies have suggested that the prognosis of CSX patients with the rate of major cardiovascular events is similar to the general population.^[6,7]^ However, in recent years, some studies have demonstrated that coronary microvascular dysfunction is a predictor of future cardiovascular events.^[8,9]^ Patients with stable angina and normal coronary arteries **increase** the **risk of** major adverse cardiovascular events, including cardiovascular death, myocardial infarction, stroke or heart failure, and all-cause mortality.^[10]^ Left ventricular longitudinal myocardial systolic function detected by speckle tracking echocardiography was significantly impaired in CSX patients,^[11]^ which is similar to ST-segment elevation myocardial infarction (STEMI).^[12]^ Also, some patients show angina attacks more frequent, prolonged, poorly responsive to medical management, depression and psychiatric disturbances, and the quality of their life is severely affected.^[13]^ On the other hand, the worsening of anginal symptoms results in angiography and repeated hospital admissions, imposing a substantial financial burden on health services.^[1,14]^

The management of patients with CSX is similar to obstructive epicardial coronary artery disease, but also different. Lifestyle modifications, cigarette quitting, blood pressure control, and cardiac rehabilitation are recommended to CSX patients.^[15,16]^ As for pharmacological treatment, the classical antianginal medications are widely used to ameliorate clinical symptoms, including β-blockers, non-dihydropyridine calcium-antagonist drugs, and nitrates. β-blockers are appropriate for patients with increased adrenergic tone. However, β-blockers used in patients with microvascular or epicardial spasm may lead to coronary vasoconstriction. Calcium channel blockers are recommended as the initial option for vasospastic angina,^[17]^ while they have no effect on improving microvascular dysfunction.^[18]^ Nitrates seem to be less effective on coronary microvascular disease due to its poor dilator effect on small resistance vessels.^[19]^ Nevertheless, the treatments above are not always based on the pathogenesis, and curative effect is not satisfactory. Thus, seeking for alternative therapies is indispensable.

Nicorandil, a potassium ATP channel opener with nitrate-like actions, recommended as a second-line treatment for stable angina by The European Society of Cardiology,^[20]^ causes epicardial coronary vasodilatation similar to nitrates, as well as dilates coronary microvessels.^[21]^ As its antianginal mechanisms correspond to the pathophysiology of coronary microvascular disease to some extent, nicorandil has been proposed as the first-choice drug for primary stable MVA in China.^[22]^ Previously clinical trials with small sample size showed nicorandil could improve symptoms in CSX patients.^[23,24]^ We conducted a meta-analysis to comprehensively evaluate the clinical curative effect and safety of nicorandil for CSX, providing more therapeutic options for patients.

## 2 Methods

This systematic review was carried out and reported following Preferred Reporting Items for Systematic Reviews and Meta-Analysis (PRISMA)^[25]^ and A Measurement Tool for the “Assessment of Multiple Systematic Reviews” (AMSTAR).^[26]^

### 2.1 Search strategy

A comprehensive search strategy was carried out including searching Pubmed (1950 to March 2020), EMBASE (1974 to March 2020), The Cochrane Library (1996 to March 2020), ClinicalTrials.gov (from inception to March 2020), China Knowledge Resource Integrated Database(CNKI)(1979 to March 2020), Chinese Science and Technique Journals Database(VIP)(1989 to March 2020), Wan Fang Database(Wan Fang)(1990 to March 2020) and the Chinese Biomedical Database(CBM)(1990 to March 2020). The following medical subject heading terms were used: “nicorandil”, “microvascular angina” and “cardiac syndrome X”.

### 2.2 Study selection

Studies meeting the following criteria were included:

1. Randomized controlled trials (RCTs);
2. Participants diagnosed as CSX by the criteria listed in Angina pectoris and normal coronary arteries: cardiac syndrome X.^[2]^ Participants with acute myocardial infarction, heart failure, hepatic failure, and renal failure were excluded;
3. The intervention was nicorandil with or without routine treatment vs controls including placebo, routine treatment, or positive medicine control. Routine treatment includes aspirin, β-blockers, angiotensin-converting enzyme inhibitors, angiotensin receptor blockers, calcium channel blockers, statins;
4. Primary outcomes including angina improvement, the resting ECG improvement, treadmill test results, readmission rate, and coronary microvascular function tests, such as coronary flow reserve (CFR) or index of microcirculatory resistance (IMR); secondary outcomes including endothelial function, and any adverse drug events/reactions (ADEs/ADRs).

There were no restrictions on the publication type and participants characteristics. Duplicate publications reporting the same groups of participants were excluded.

The titles, abstracts, and keywords of records retrieved were scanned to determine whether to be assessed further. Full articles were retrieved for further assessment if the information met the inclusion criteria. Any disagreement between reviewers was resolved by discussion or consulting a third party.

### 2.3 Data extraction and management

Data concerning details of the study population, intervention and outcomes were extracted independently by 2 reviewers. For binary outcomes, the number of events and total number in each group was extracted. For continuous outcomes, mean, standard deviation and sample size of each group were extracted. The data extraction form included the following items:

(1) General information: title, authors, and year of publication.
(2) Trial characteristics: study design, method of randomization, allocation concealment, blinding.
(3) Patients: number in treatment and control groups, age, diagnostic criteria, withdrawals/losses to follow-up (reasons/description), subgroups.
(4) Intervention: intervention (dose, course of treatment, and frequency), comparison intervention (dose, course of treatment, and frequency).
(5) Outcomes: outcomes specified above. The study was not conducted directly on patients, therefore ethical approval was not necessary.

### 2.4 Quality assessment

The methodological quality of trials was assessed independently using criteria from the Cochrane Handbook for Systematic Review of Interventions, Version 5.1.0.^[27]^ Seven domains are considered such as sequence generation (selection bias), allocation concealment (selection bias), blinding of participants and personnel (performance bias), blinding of outcome assessment (detection bias), incomplete outcome data (attrition bias), selective outcome reporting (reporting bias), and other bias. Three levels of “low risk”, “high risk”, or “unclear risk” were the quality appraisal category. Any disagreements were resolved by mutual consensus.

### 2.5 Data synthesis

Revman 5.3 software provided by the Cochrane Collaboration was used for data analyses. The model used to pool the data depends on the existence and extent of heterogeneity. If the *I*^2^ statistics were less than 50%, the heterogeneity could be accepted, and the fixed-effect model was chosen. If the *I*^2^ statistics exceeded 50%, the random-effects model was used. When heterogeneity among studies was obvious (*I*^2^ > 50%), the sources of heterogeneity would be investigated. For binary outcomes, the pooled relative risk (RR) with 95% confidence interval (CI) was used as the effect measure. For the continuous outcome, weighted mean differences (WMD) or standardized mean differences (SMD) was used as the effect measure, both with 95% confidence intervals (CI). The approach to incorporating cross over trials is to take all measurements from the 2 intervention periods and analyse these as if the trial were a parallel-group trial.^[27]^ Publication bias would be assessed by funnel plot and the Eggers test if the group included more than 10 studies. The Eggers test was performed by software Stata 14.0 (Stata Corp, College Station, Tex).

### 2.6 Subgroup analysis, sensitivity analysis, and meta-regression

Subgroup analysis, sensitivity analysis, and meta-regression analysis were performed to explore potential sources of heterogeneity. Subgroup analysis was also conducted to determine whether there was a different effect of an intervention in different situations. Sensitivity analysis was completed by changing the effect model or removing 1 study at a time to investigate the influence of a single study on the overall pooled estimate. Meta-regression analysis was performed using residual maximum likelihood (REML) with Knapp-Hartung modification by software Stata 14.0.

### 2.7 Quality of evidence assessment

The quality of evidence was assessed using The Grading of Recommendations Assessment, Development and Evaluation (GRADE) approach^[28]^ by which a determination of high, moderate, low, or very low was made for each major outcome.

## 3 Results

**Figure 1:**
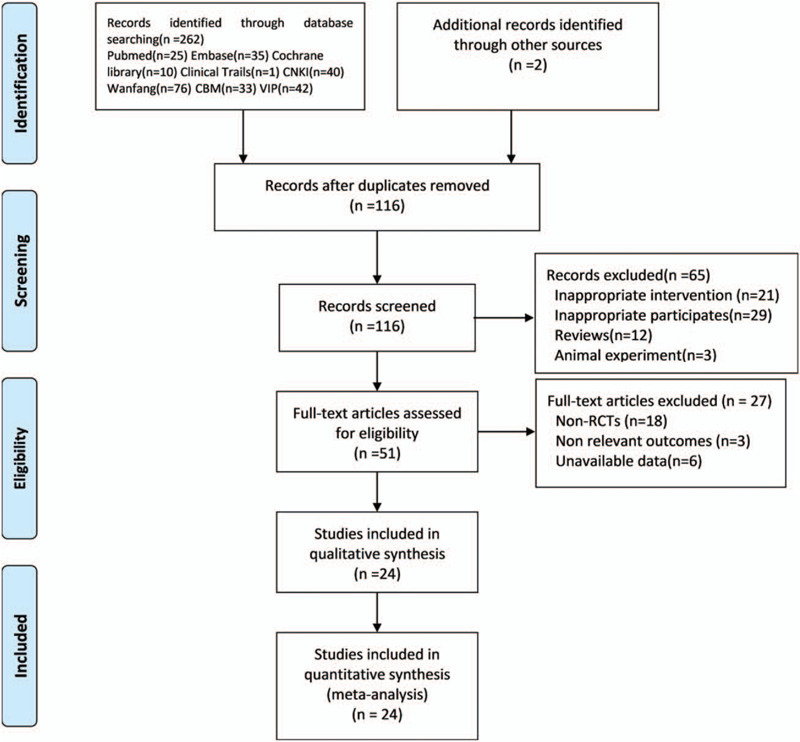
Flow diagram of study selection. CBM = Chinese Biomedical Database, CNKI = China knowledge resource integrated database, RCTs = randomized controlled trials, VIP = Chinese Science and Technique Journals Database.

In total, 264 records were identified. After duplicates among different databases were removed, 116 records were kept for further assessment. The full texts of 51 remaining records were downloaded for careful assessment. There were 24 trials included in the review. The detailed process of search and identification was shown in Figure 1.

Details of the 24 included studies^[24,29–51]^ are shown in Table 1. Twenty two studies^[30–51]^ included were parallel test, and 2 studies^[24,29]^ were cross over design. Trial duration ranged from 3 weeks to 6 months. The number of participants in the studies ranged from 13 to 274, with a total of 2323participants included in this review. The average ages of participants ranged from 43 to 64 years old.

**Table 1:**
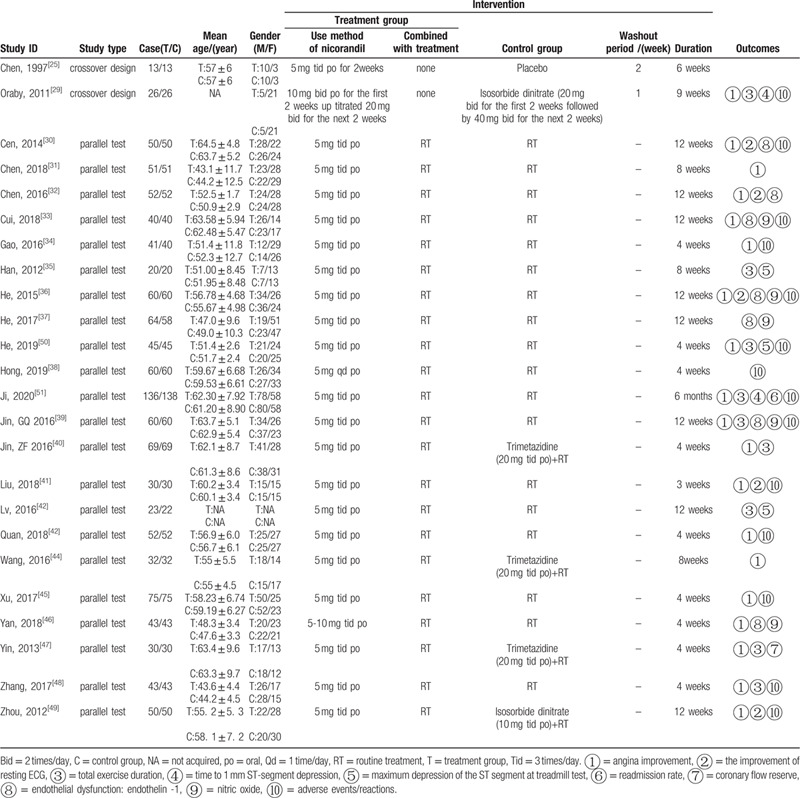
Characteristics of included studies.

One study^[24]^ compared nicorandil with a placebo. One study^[29]^ compared nicorandil with isosorbide dinitrate. One study^[49]^ made a comparison between nicorandil with routine treatment and isosorbide dinitrate with routine treatment. Three studies^[40,44,47]^ compared between nicorandil with routine treatment and trimetazidine with routine treatment. The other studies^[30–39,41–43,45,46,48,50,51]^ compared nicorandil with routine treatment vs routine treatment.

Nicorandil was given by oral administration. The dose of nicorandil varied across studies: 5 mg once a day used in 1 study,^[38]^ 10 mg twice a day for the first 2 weeks, then increasing to 20 mg twice a day for the next 2 weeks used in 1 study,^[29]^ 5 mg 3 times a day used in 21 studies,^[24,30–37,39–45,47,48,50,51]^ 5 to 10 mg 3 times a day used in 1 study.^[46]^

The patients in 10 studies^[34,38,40,41,43,45–48,50]^ received treatment for less than or equal to 4 weeks, and the patients in 5 studies^[24,29,31,35,44]^ received treatment for 6 to 9 weeks, and the remaining patients in 8 studies^[30,32–34,36,37,39,49]^ received treatment for 12 weeks, the patients in 1 study^[51]^ received treatment for 6 months.

Twenty studies^[24,29–34,36,39–41,43–51]^ reported rate of angina improvement. Six studies^[30,32,36,39,41,49]^ reported the improvement of resting ECG. In regard to treadmill exercise test, 9 studies^[24,29,35,40,42,47,48,50,51]^ reported total exercise duration during. Three studies^[24,29,51]^ reported the time to 1 mm ST-segment depression. Four studies^[24,35,42,50]^ compared maximum depression of the ST-segment at treadmill exercise test. Only 1 study^[51]^ reported the readmission rate. One study reported CFR.^[47]^ Seven studies^[30,32,33,36,37,39,46]^ reported the level of ET-1 and NO.

Fifteen studies^[24,29,30,33,34,36,38,39,41,43,45,48–51]^ observed ADEs/ADRs, thirteen^[24,29,30,33,36,38,39,41,43,45,48,49,51]^ of them reported positive result. The details of the study characteristics were summarized in Table 2.

**Table 2:**
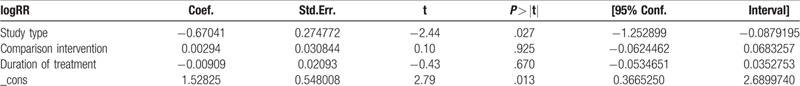
Meta-regression of basic characteristics of RCTs and RRs of angina improvement.

### 3.1 Methodological quality

Firstly, the risk of bias in cross over trials is assessed according to Cochrane Handbook. CSX is a condition that is chronic and relatively stable. The primary outcomes do not include irreversible conditions, that is, death. The carryover effect contains a pharmacological effect and psychological effect. In Chens^[24]^ and Orabys^[29]^ studies, random, double-blinding, and a washout period between treatment periods may reduce the risk of carryover effect. None of the participants dropped out after the first treatment, and all of them finished the two-stage treatment. Both the cross over studies adopted two-stage data. But the authors did not offer paired data, and neither of them mentioned the randomization method or allocation concealment. One study^[24]^ reported withdrawal, but the intention-to-treat (ITT) analysis was not mentioned.

**Figure 2:**
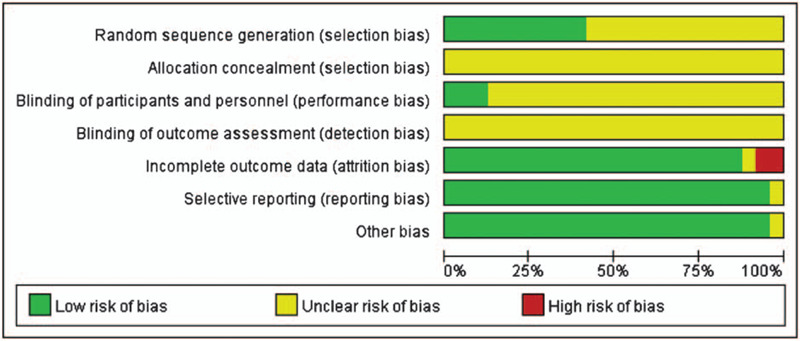
Risk of bias graph: review authors judgements about each risk of bias item presented as percentages across all included studies.

**Figure 3:**
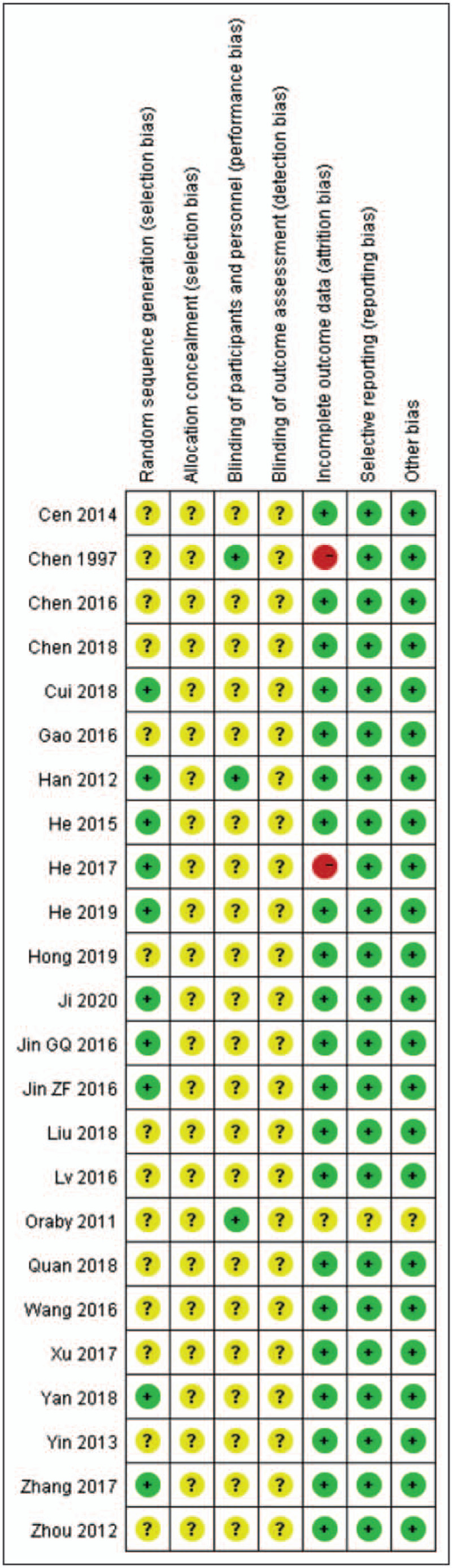
Risk of bias summary: review authors judgements about each risk of bias item for each included study.

All the parallel-group trials^[30–51]^ studies mentioned randomization. However, only 9 studies^[30,33,35–37,46,48,50,51]^ described the allocation sequence being generated from random number tables, and 1 study^[39]^ elaborated by sortition. One study^[37]^ mentioned withdrawal. None mentioned allocation concealment or ITT analysis. Only 1 study^[35]^ mentioned single blinding. We believed all included studies to be free of selective reporting because the same outcomes were described in the methods and reported in the results. In all studies, the characteristics of participants in different treatment groups were similar at baseline (age, sex, the severity of angina). So we considered all included trials to be free of other potential sources of bias (Figs. 2 and 3).

### 3.2 Publication bias

**Figure 4:**
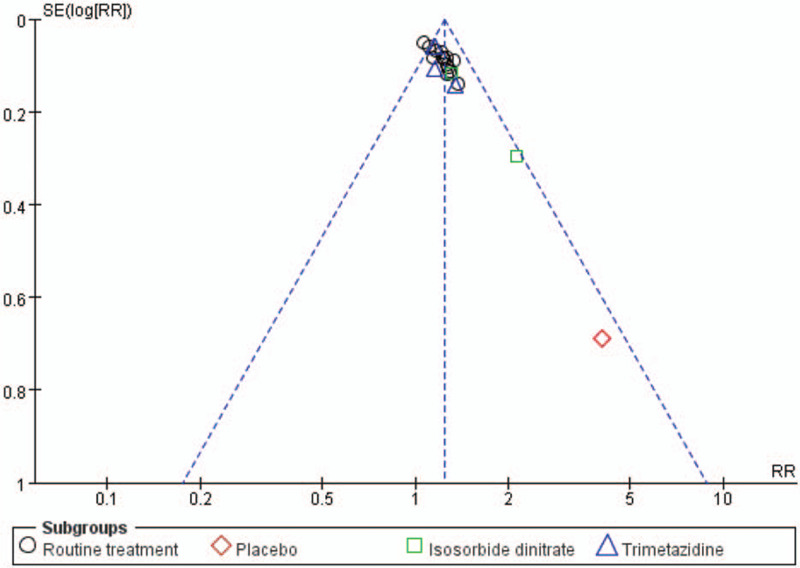
Funnel plot of publication bias according to the rate of angina symptoms improvement.

**Figure 5:**
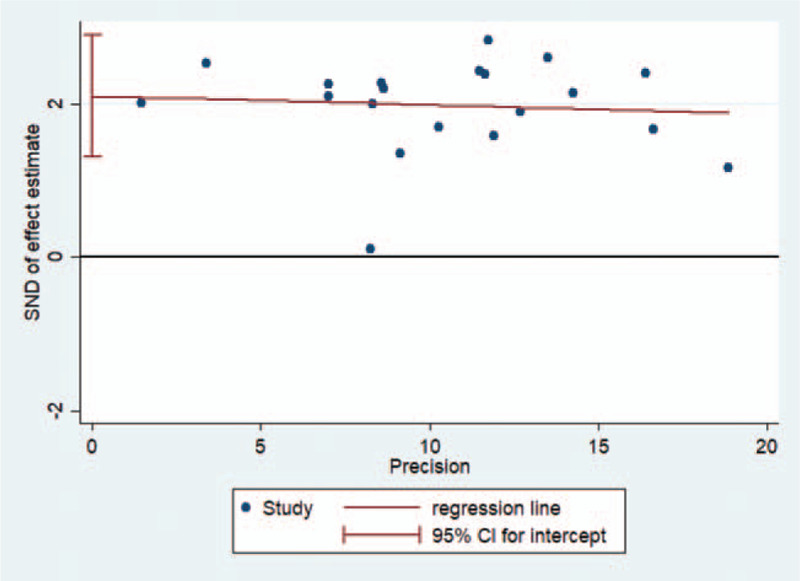
Eggers test for evaluating the publication bias in the studies of angina symptoms improvement.

The funnel plot was slightly asymmetric when pooling 20 trials on the rate of angina symptoms improvement, which indicated some evidence of publication bias (Fig. 4). The Eggers test showed that significant statistical publication bias was detected (Eggers test *P* = .000) (Fig. 5).

### 3.3 Effects of interventions

#### 3.3.1 Angina symptoms improvement: rate of angina symptoms improvement

**Figure 6:**
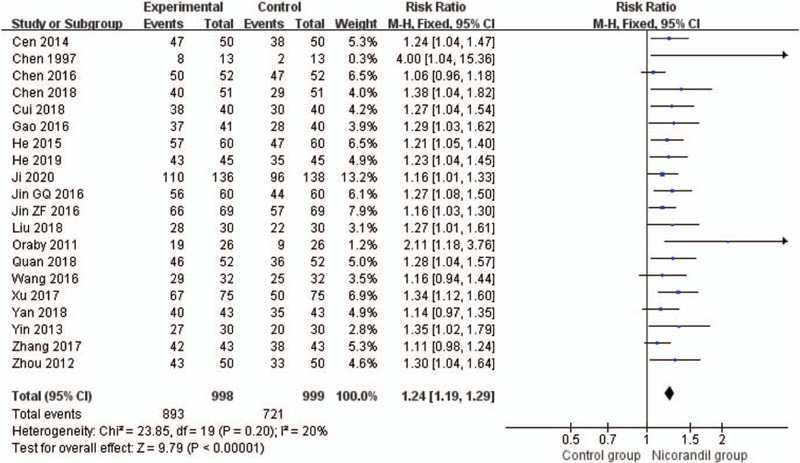
Forest plot of rate of angina symptoms improvement.

**Figure 7:**
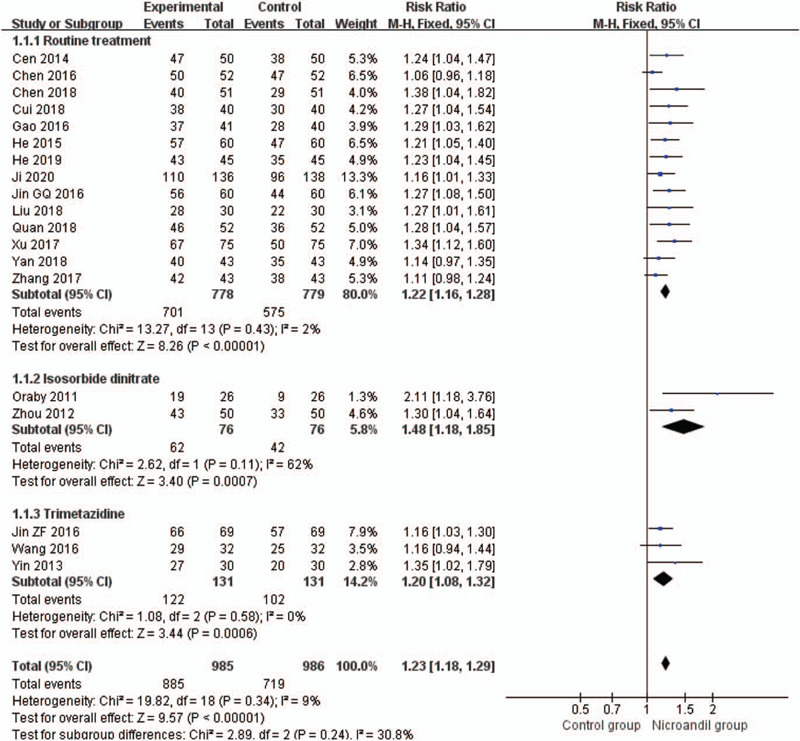
Forest plot of rate of angina symptoms improvement, subgroup analysis according to comparison intervention.

Twenty studies^[24,29–34,36,39–41,43–51]^ assessed the rate of angina symptoms improvement. A decrease in the frequency of angina attacks was the measure. Nicorandil has a better effect on improving angina symptoms (RR 1.24, 95% CI 1.19 to 1.29, *I*^2^ = 20%, *P* < .00001, Fig. 6). Subgroup analysis shows the effects of treatment group on improving angina symptoms are better than routine treatment alone^[30–34,36,39,41,43,45,46,48,50,51]^ (RR 1.22, 95% CI 1.16 to 1.28, *I*^2^=2%, *P* < .00001, Fig. 7), trimetazidine with routine treatment^[40,44,47]^ (RR 1.20, 95% CI 1.08–1.32, *I*^2^ = 0%, *P*=.0006, Fig. 7), and isosorbide dinitrate group^[29,49]^ (RR 1.48, 95% CI 1.18–1.85, *I*^2^=62%, *P* = .0007, Fig. 7). There is only 1 RCT^[24]^ in placebo group, synthesis analysis cannot be performed.

**Figure 8:**
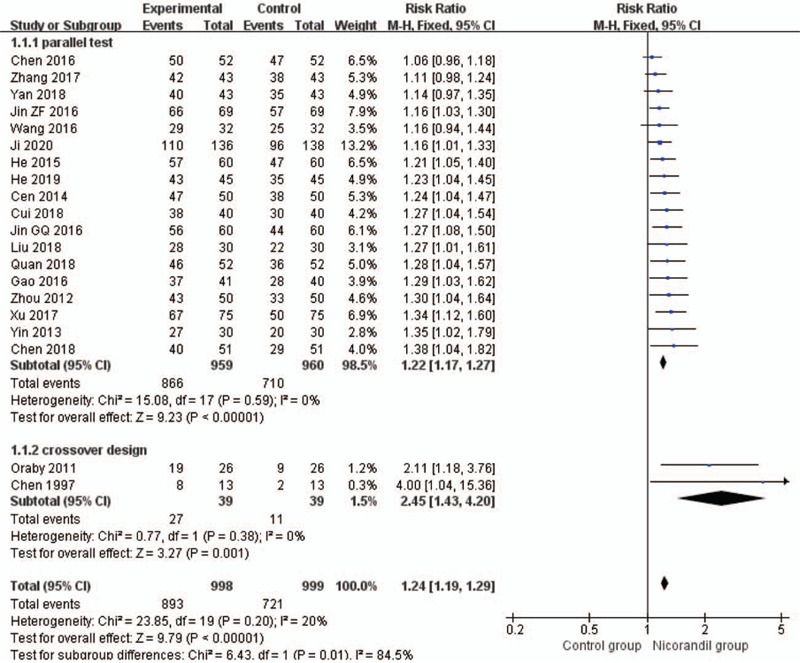
Forest plot of rate of angina symptoms improvement, subgroup analysis according to the study type.

To explore this heterogeneity, we conducted a meta-regression analysis of study type, comparison intervention, and duration of treatment by REML with Knapp-Hartung modification. Tau^2^ was equal to 0.001136 as REML estimates of between-study variance. *I*^2^ was 0.00% in terms of the proportion of residual variation due to heterogeneity. The adjusted *R*^2^ value was equal to −45.61%, with the proportion of between-study variance explained. Data from Table 2 shows there was a significant association between study type and the final result (*P* = .027, 95% CI −1.25 to −0.087). We divided the studies into 2 subgroups according to study type, and then the subgroup analysis showed no statistical heterogeneity was found in each subgroup (*P* = .59, *I*^2^ = 0%; *P* = 0.38, *I*^2^ = 0%, Fig. 8).

#### 3.3.2 Resting ECG improvement: rate of resting ECG improvement

**Figure 9:**
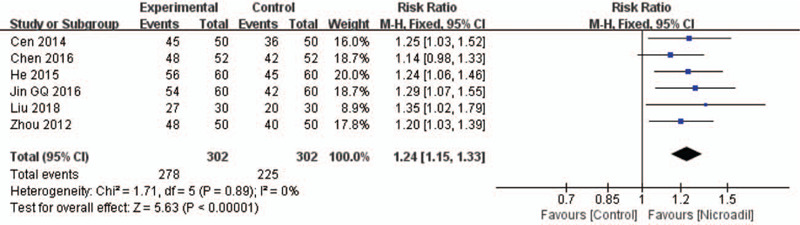
Forest plot of rate of resting ECG improvement. ECG = Electrocardiogram.

Six studies^[30,32,36,39,41,49]^ reported the rate of ECG improvement. Improvement of ST-segment depression and T wave inversion was the most common measure. Nicorandil combined with routine treatment has a better effect on improving ECG compared with the control group (RR = 1.24, 95% IC: 1.15–1.33, *I*^2^ = 0%, *P* < .00001, Fig. 9).

#### 3.3.3 Treadmill test result: total exercise duration

**Figure 10:**
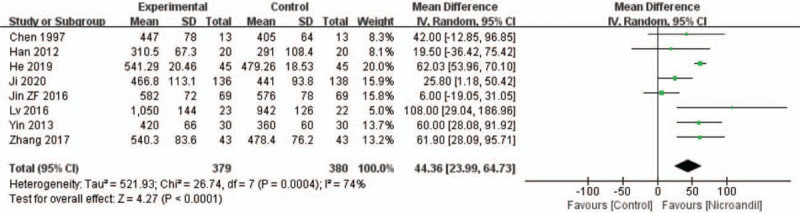
Forest plot of total exercise duration.

Nine studies^[24,29,35,40,42,47,48,50,51]^ reported total exercise duration. Available data could be extracted from 8 studies.^[24,35,40,42,47,48,50,51]^ Because obvious heterogeneity was observed among these studies, a random-effect model was used. The result showed that nicorandil had a better effect on increasing total exercise duration than the control group (WMD = 44.36, 95% IC: 23.99–64.73, *I*^2^ = 74%, *P* < .0001, Fig. 10). We removed the Jin ZF 2016 study, the heterogeneity in the 7 remaining studies is moderate. Meta-analysis of these 7 studies showed the effect of nicorandil on increasing total exercise duration remained(WMD = 51.98, 95% IC: 35.85–68.10, *I*^2^ = 47%, *P* < .00001).

#### 3.3.4 Treadmill test result: time to 1 mm ST-segment depression

**Figure 11:**
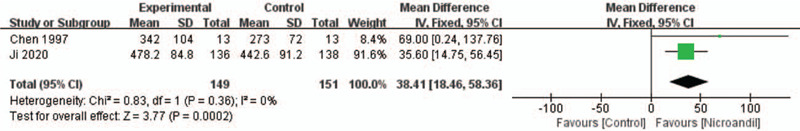
Forest plot of time to 1 mm ST-segment depression.

Three studies^[24,29,51]^ reported time to 1 mm ST-segment depression, but available data only could be extracted from 2 studies.^[24,51]^ The result showed that nicorandil had a better effect on prolonging time to 1 mm ST-segment depression (WMD =38.41, 95% IC: 18.46–58.36, *I*^2^ = 0%, *P* = .0002, Fig. 11).

#### 3.3.5 Treadmill test result: maximum depression of the ST-segment

**Figure 12:**
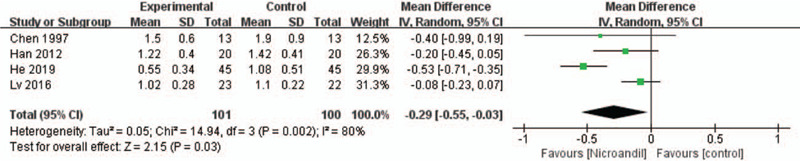
Forest plot of maximum depression of the ST-segment.

Four studies^[24,35,42,50]^ reported maximum depression of the ST-segment. A random-effect model was applied because of the obvious heterogeneity. The result showed that nicorandil had a better effect on improving the maximum depression of the ST-segment (WMD =−0.29, 95% IC: −0.55 to −0.03, *I*^2^ = 80%, *P* = .03, Fig. 12). When we removed the He 2019 study, there was no evidence of heterogeneity in the remaining studies. However, meta-analysis demonstrated no significant difference between the treatment group and control groups in the maximum depression of the ST-segment (WMD = −0.12, 95% IC: −0.25 to 0, *I*^2^ = 0%, *P* = .05).

#### 3.3.6 Readmission rate

Only 1 study^[51]^ reported the readmission rate. The rate of readmission in the nicorandil group (22/136) was lower than in the control group (38/138).

#### 3.3.7 Coronary microvascular function test: coronary flow reserve

Only 1 study^[47]^ reported CFR measured by transthoracic Doppler echocardiography. Nicorandil combined with routine treatment had an advantage of increasing CFR than trimetazidine combined with routine treatment (WMD = 0.36, 95% IC: 0.07–0.65, *P* = .01).

#### 3.3.8 Endothelial function: the level of endothelin-1

**Figure 13:**
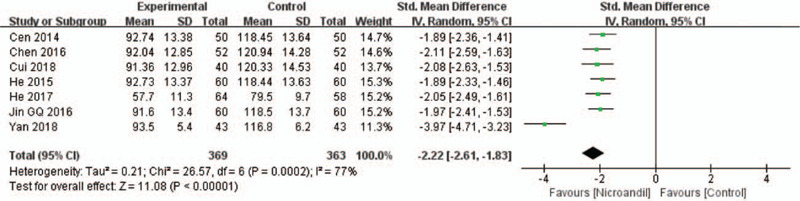
Forest plot of ET-1 level. ET-1 = Endothelin-1.

Seven studies^[30,32,33,36,37,39,46]^ reported changes in endothelin-1 (ET-1) levels. Statistical heterogeneity was observed, and the units of outcomes varied. Thus, a random-effect model and SMD were used. Pooled results indicated greater effects of nicorandil on reducing ET-1 levels (SMD = −2.22, 95% IC: −2.61 to −1.83, *I*^2^ = 77%, *P* < .00001, Fig. 13). When we removed the Yan 2018 study, there was no evidence of heterogeneity in the remaining studies, and meta-analysis showed the effect remained (SMD = −1.99, 95% IC: −2.18 to −1.8, *I*^2^ = 0%, *P* < .00001).

#### 3.3.9 Endothelial function: the level of nitric oxide

**Figure 14:**
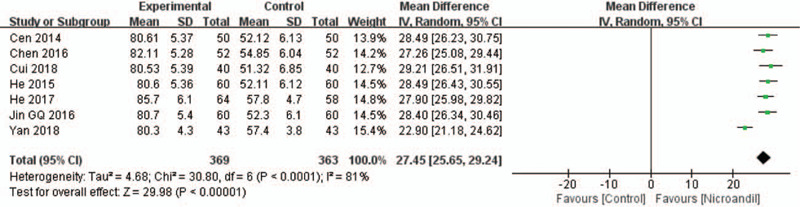
Forest plot of NO level. NO = Nitric oxide.

Seven studies^[30,32,33,36,37,39,46]^ reported changes in nitric oxide (NO) levels. Statistical heterogeneity was observed, thus, a random-effect model was used. Pooled results indicated greater effects of nicorandil on increasing NO levels (WMD = 27.45, 95% IC: 125.65–29.24, *I*^2^ = 81%, *P* <.00001, Fig. 14). When we removed the Yan 2018 study, there was no evidence of heterogeneity in the remaining studies, and meta-analysis showed the effect remained (WMD = 28.22, 95% IC: 27.34–29.11, *I*^2^ = 0%, *P* < .00001).

### 3.4 Adverse events/reactions

Among the 24 included studies, 15 studies observed ADEs/ADRs,^[24,29,30,33,34,36,38,39,41,43,45,48–51]^ 13 studies^[24,29,30,33,36,38,39,41,43,45,48,49,51]^ reported positive results. Headache, dizziness, and gastrointestinal symptom were the major adverse drug reactions in both treatment group and control group. The details of ADEs/ADRs were summarized in Table 3.

**Table 3:**
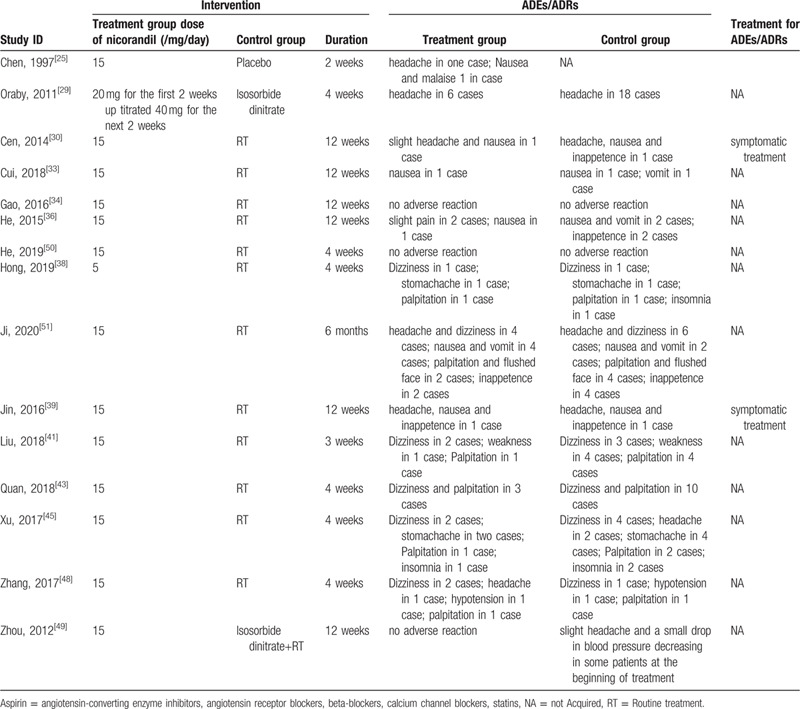
Adverse events/reactions.

### 3.5 Quality of evidence assessment

The overall assessment of quality of evidence ranged from very low to low according to GRADE methodology, which was summed up in Table 4.

**Table 4:**
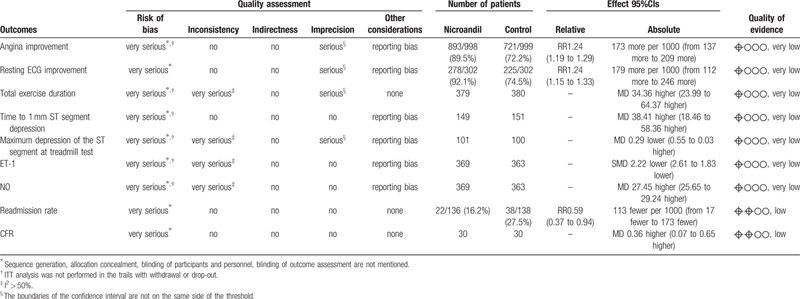
GRADE evidence profile.

## 4 Discussion

### 4.1 Findings

Angina symptoms improvement, resting ECG improvement, treadmill test result, and endothelial function have become the main outcomes analyzed in this review. The results of our meta-analysis showed that nicorandil had a better effect on improving angina symptoms, decreasing the frequency of angina attack, and improve ST-segment depression and T wave inversion in resting ECG. The treadmill test can be used to evaluate the efficacy of therapy on control of ischemia.^[20]^ The effect of nicorandil on increasing total exercise duration and prolonging time to 1 mm ST-segment depression was better than the control group. However, there was a large heterogeneity between the 2 groups in total exercise duration. We performed a sensitivity analysis by removing 1 study^[40]^ with trimetazidine as comparison intervention and found that the effect of nicorandil on increasing total exercise duration remained. Also, the apparent heterogeneity in the maximum depression of the ST-segment attributed to the baseline levels before treatment. We removed the study,^[50]^ whose maximum ST-segment depression was less than the others, and found heterogeneity disappeared. However, there was no statistically significant difference between the nicorandil group and control group. Therefore, the effect on improving the maximum depression of the ST-segment for nicorandil is not definite. Recurrent angina attacks contribute to repeated multiple diagnostic investigations and hospitalization, which impaired the quality of life. Nicorandil may lower down the rehospitalization rate of patients with CSX, but the result needs more studies to confirm.

The pathogenesis of CSX is attributed to CMD, which is defined as CFR < 2.0 measured by positron emission computed tomography.^[8]^ CFR refers to the ratio of myocardial blood flow during near maximal coronary vasodilatation to baseline myocardial blood flow.^[52]^ Decreased CFR is representative of microvascular dysfunction.^[53]^ Moreover, low CFR has predicted a poor prognosis in patients with and without obstructive CAD.^[54]^ However, only 1 study reported that nicorandil had an advantage of increasing CFR, hence there is insufficient evidence to support the effect of nicorandil on improving CFR. Endothelial dysfunction is the most accepted mechanism leading to CMD, can be defined as an imbalance between vasodilator factors such as NO, prostacyclin, and vasoconstrictor factors such as ET-1, thromboxane A2, prostaglandin H2.^[55]^ Reduced bioavailability of NO influences the migratory and angiogenic of endothelial cells, inducing vessel destruction, microvascular rarefaction, and decreased microvascular density, which may partly explain the coronary microvasculature abnormalities in patients with coronary microvascular disease.^[56,57]^ The concentration of NO and the NO/ET-1 ratio are decreased in patients with CSX.^[58]^ Our study showed that the ET-1 level was decreased, and the NO level was significantly increased by nicorandil. The sensitivity analysis was further performed for ET-1 with obvious heterogeneity. We removed 1 study^[46]^ with a 4 weeks treatment and found that the effect of nicorandil on reducing the level of ET-1 was not significantly changed. Large heterogeneity also was observed among the studies on NO level. We excluded the study^[46]^ with a 4 weeks treatment and found that the effect of nicorandil on increasing NO level remained. We can speculate that the effect of nicorandil on ET-1 and NO level is associated with the duration of treatment.

It has been reported that nicorandil could augment CFR in patients with angina pectoris and nearly normal coronary arteriograms.^[23]^ Studies have shown that intracoronary nicorandil ameliorated microvascular dysfunction, which was evaluated with IMR, and improved CFR in patients with STEMI undergoing primary percutaneous coronary intervention.^[59,60]^ In addition, nicorandil may improve chest pain symptoms, and regulate plasma NO and ET-1 in the coronary slow flow.^[61]^

Nicorandil as a vasodilator exerts effects both as a potassium ATP channel opener and a nitrate.^[21]^ It can dilate the coronary artery microvessels with a diameter of <100 μm, thus reduce coronary arterial resistance, causing an increase in coronary blood flow.^[62]^ It also enhances ischemic preconditioning through the activation of the potassium ATP channel in mitochondrial membranes.^[63]^ Oxidative stress is associated with impaired endothelium. Several pieces of research have reported that there is systemic oxidative stress in cardiac syndrome X patients.^[64,65]^ Nicorandil decreases xanthine oxidase-generated reactive oxygen species induced by rapamycin. Moreover, it can increase reendothelialization impaired by rapamycin and endothelial nitric oxide synthase expression inhibited by rapamycin.^[66]^

There is inadequate reporting on ADEs/ADRs in the included trials. Known ADRs of nicorandil mainly were headache, nausea and vomit, dizziness, fever, weakness, ulceration, liver dysfunction, jaundice, thrombopenia. Some ADRs in this review we found were new, for instance, palpitation, stomachache, inappetence, insomnia, hypotension, and so on. They were not serious and relieved by symptomatic treatment. We could not make a clear causal connection judge due to nicorandil or other routine treatments. Some case reports mentioned ulcers induced by nicorandil, including oral, anal, perianal, perivulval, gastrointestinal, colonic, peristomal and skin ulceration,^[67,68]^ which were not found in these including studies.

Most of the included studies were classified as being low quality, and they were assessed as having an unclear risk of bias with the Cochrane Collaboration “Risk of bias” tool. The sample size calculation was not reported in any study. ITT analysis was not performed in the trails with withdrawal or drop-out. Only 1 study^[24]^ used a placebo as a control treatment. Whats more, no multi-center, large scale RCT was found. All studies are small, with positive findings, and without ITT analysis, resulting in publication bias. Hence, we need more high-quality RCTs to prove the efficacy and safety of nicorandil for CSX patients.

We found out 2 systematic reviews of CSX. One review^[69]^ evaluated the efficacy of traditional Chinese medicine, including Chinese patent drug, decoction, and Chinese medicine injection, compared with conventional treatment for patients with CSX. Another review^[70]^ assessed the efficacy and safety of Tongxinluo Capsule for CSX. Their outcomes were angina symptom improvement, ECG improvement, treadmill test results, and ET-1 level, lacking readmission rate and coronary microvascular function test.

### 4.2 Implications for practice

There is very low to low-quality evidence from the included studies to suggest that nicorandil is not an effective intervention for patients with CSX. More high-quality studies are required to identify its efficacy and safety. The prescription drug label of nicorandil states that it is used for angina pectoris, but there is no restriction on the type of angina. It is necessary to identify the mechanism of nicorandil for CSX by more experiments and clinical researches.

### 4.3 Implications for future research

The methodological quality of clinical trials of treatment with nicorandil for CSX needs to be improved. Firstly, methods of random sequence generation and allocation concealment should be described, blinding, and sample size calculation should be applied in the study. Secondly, clinical trial registries should be encouraged to provide the available protocol. Thirdly, if participants withdraw or drop out of the study, the ITT analysis should be performed.

The design of future clinical trials also should be more perfect. Comparison intervention being given a placebo can make sure the clinical effect. The implementation of long-term follow-up is necessary to evaluate prognosis. Clinically relevant outcomes should be reported, such as CFR, IMR, readmission rate, and adverse cardiovascular events. Also, pharmacoeconomics analysis can be applied to optimize the therapeutic schedule.

### 4.4 Limitations

There were some potential limitations in our systematic review:

1. Just English and Chinese databases were searched because of the language barrier.
2. The methodological quality of these included studies was of low quality.
3. The sample sizes of the present studies were small, which may lead to bias.
4. The longest period of follow-up among the included trials in our research was just 6 months, thus we could not identify the long-term effect of nicorandil on CSX.
5. There was significant statistical heterogeneity for treadmill test results and endothelial function.

We concluded the possible explanations for the apparent heterogeneity were comparison intervention, duration of treatment. Therefore, the results should be interpreted with caution.

## 5 Conclusions

Nicorandil appears to have some benefit on improving angina symptoms, resting ECG, treadmill test result, ameliorating endothelial dysfunction, and also seems to be relatively safely used in clinical. Due to the low methodological quality of the RCTs, the risk of publication bias, and significant statistical heterogeneity, there is insufficient evidence for the efficacy or safety of nicorandil in the treatment of CSX. The results from this review still need larger, well designed, and high-quality trials to confirm.

## Author contributions

**Conceptualization:** Yuanhui Hu.

**Data curation:** Guozhen Yuan, Jingjing Shi.

**Formal analysis:** Qiulei Jia, Guozhen Yuan.

**Methodology:** Qiulei Jia, Shuqing Shi, Jingjing Shi, Shuai Shi, Yi Wei, Yuanhui Hu.

**Visualization:** Shuqing Shi, Shuai Shi.

**Writing – original draft:** Qiulei Jia.

**Writing – review & editing:** Qiulei Jia, Shuqing Shi, Yi Wei, Yuanhui Hu.
